# Die Alkoholabhängigkeit im Spannungsfeld der Geschlechterrollen

**DOI:** 10.1007/s00115-025-01904-9

**Published:** 2025-09-29

**Authors:** Eva Friedel, Nina Müller-Wirth, Pichit Buspavanich, Maximilian Berger, Pauline Meurer, Philine Claussen, Julie L. O’Sullivan, Sandy Doll, Frank Schulte-Derne, Ingar Abels

**Affiliations:** 1 Klinik für Psychiatrie und Psychotherapie, Charité – Universitätsmedizin Berlin, Charitéplatz 1, 10117 Berlin, Deutschland; 2 Forschungsbereich Mind and Brain, Klinik für Psychiatrie und Psychotherapie, Charité – Universitätsmedizin Berlin, Charitéplatz 1, 10117 Berlin, Deutschland; 3 Arbeitsbereich Geschlechterforschung in der Medizin, Charité – Universitätsmedizin Berlin, Augustenburger Platz 1, 13353 Berlin, Deutschland; 4 Institut für Sexualwissenschaft und Sexualmedizin, Charité – Universitätsmedizin Berlin, Charitéplatz 1, 10117 Berlin, Deutschland; 5 Institut für Medizinische Soziologie und Rehabilitationswissenschaft, Charité – Universitätsmedizin Berlin, Charitéplatz 1, 10117 Berlin, Deutschland; 6 Standort Berlin-Potsdam, Deutsches Zentrum für Psychische Gesundheit, Berlin, Deutschland; 7 Landschaftsverband Westfalen-Lippe, LWL-Koordinationsstelle Sucht, Schwelingstaße 11, 48145 Münster, Deutschland; 8 Stellvertretende Zentrale Frauen- und Gleichstellungsbeauftragte, Charité Universitätsmedizin Berlin, Charitéplatz 1, 10117 Berlin, Deutschland

**Keywords:** Alkoholbezogene Störungen, Geschlecht, Suchtmedizin, Maskulinität, Gewalt in der Partnerschaft, Alcohol use disorders, Gender, Addiction medicine, Masculinity, Intimate partner violence

## Abstract

Die vorliegende Arbeit beleuchtet den Einfluss von Geschlechterrollen auf die Entstehung, den Verlauf und die Versorgung alkoholbezogener Störungen. Epidemiologische Daten zeigen signifikante Unterschiede zwischen den Geschlechtern hinsichtlich Prävalenz, Konsummuster und Komorbiditäten bei Alkoholabhängigkeit. Diese Unterschiede sind nicht nur biologisch bedingt, sondern spiegeln auch soziale Konstruktionen von Geschlecht und geschlechtsspezifische Rollenerwartungen wider. Traditionelle Männlichkeitsnormen sind dabei mit erhöhtem Alkoholkonsum, geringerer Inanspruchnahme von Hilfsangeboten und höherer Gewaltbereitschaft assoziiert, während Frauen häufiger von internalisierenden psychischen Erkrankungen sowie von gesellschaftlicher Stigmatisierung betroffen sind. LGBTIQ*-Personen sind zusätzlich aufgrund von Minderheitenstress und strukturellen Barrieren besonders vulnerabel. Der Beitrag fordert eine geschlechtersensible Suchthilfe, die psychosoziale Belastungen, Gewalt- und Traumafolgen sowie strukturelle Ungleichheiten adressiert. Ein besonderer Fokus liegt auf der kritischen Auseinandersetzung mit rigiden Geschlechterrollen, die sowohl problemverstärkend als auch behandlungserschwerend wirken können.

**Zusatzmaterial online:**

Die Online-Version dieses Beitrags (10.1007/s00115-025-01904-9) enthält zwei Tabellen.

## Abstract (en)

This article explores the impact of gender roles on the development, course, and treatment of alcohol use disorders. Epidemiological data reveal significant gender differences in prevalence, patterns of use, and comorbidities related to alcohol dependence. These differences are not solely biologically determined but also reflect the social construction of gender and culturally specific role expectations. Traditional masculinity norms are associated with increased alcohol consumption, reduced help-seeking behavior, and greater propensity for violence, while women are more likely to experience internalizing mental illnes and societal stigma. LGBTIQ* individuals are particularly vulnerable due to minority stress and structural barriers. This article advocates for gender-sensitive addiction care that addresses psychosocial stressors, trauma, and systemic inequalities. It emphasizes the need to critically examine rigid gender roles, which may exacerbate problems and hinder effective treatment.

## Hintergrund

Zahlreiche epidemiologische Studien belegen geschlechterspezifische Unterschiede in der Prävalenz, im Konsummuster sowie im Verlauf und in den Komorbiditäten alkoholbezogener Störungen. Diese Unterschiede sind nicht ausschließlich biologisch erklärbar, sondern spiegeln komplexe Wechselwirkungen zwischen biologischen und sozialen Faktoren und damit auch geschlechterspezifischen Rollenerwartungen wider.

Bislang beziehen sich die meisten verfügbaren Daten nur auf die binären Geschlechtskategorien Mann und Frau: Männer weisen demnach weltweit eine signifikant höhere Prävalenz von Alkoholabhängigkeit („alcohol use disorder“, AUD) auf als Frauen und sterben häufiger an alkoholbedingten Ursachen. 75 % der ambulant betreuten Suchtkranken in Deutschland sind Männer, bei der Hälfte der Betroffenen ist Alkohol die Hauptsubstanz [30]. Männer konsumieren Alkohol häufiger in sozialen Kontexten, die mit Risikoverhalten assoziiert sind, während Frauen mit Alkoholabhängigkeit in höherem Maße internalisierende Komorbiditäten wie Depressionen, posttraumatische Belastungsstörung (PTBS) und Angststörungen aufweisen [16]. Studien zeigen zudem, dass die Prävalenz der AUD bei Frauen zunimmt [33], die Erkrankung bei Frauen häufig schwerwiegender verläuft und dass die gesundheitlichen Folgen trotz geringerer Konsummengen deutlich erhöht sind [28].

Weitergedacht müssen hierbei auch LGBTIQ*^1^-Personen Beachtung finden: Aktuelle Untersuchungen weisen auf deutlich erhöhte Prävalenzraten für Substanzkonsum und -störungen in dieser Gruppe hin. Flentje und Kolleg*innen [10] zeigten in einer groß angelegten Metaanalyse, dass trans* und genderdiverse Personen signifikant häufiger Alkohol und andere Substanzen konsumieren als cis-geschlechtliche^2^ Vergleichsgruppen, was unter anderem auf Minderheitenstress, Diskriminierung und erschwerten Zugang zu Gesundheitsdiensten zurückgeführt wird [27].

Die in diesem Beitrag besprochenen Arbeiten wurden nach dem Ermessen der Autor*innen ausgewählt. Es wurde nicht versucht, Studien oder Daten aus bestehenden klinischen oder epidemiologischen Untersuchungen systematisch zusammenzufassen. Insbesondere haben wir keine systematische Literaturrecherche durchgeführt, um alle relevanten Studien zum Thema Geschlecht, Gender und Alkoholabhängigkeit zu identifizieren. Ebenso wurden keine statistischen Tests angewendet. Umfassende systematische Übersichtsarbeiten zu diesem Themenfeld liegen beispielsweise vor von Cook et al. [6] und anderen [14, 22, 35].

Vor dem Hintergrund dieser epidemiologischen und sozialen Unterschiede stellt sich die Frage, wie „Geschlecht“ in der Forschung und klinischen Praxis überhaupt verstanden und erfasst wird. Eine differenzierte Betrachtung ist notwendig, um sowohl biologische als auch psychosoziale Dimensionen zu berücksichtigen.

## Geschlecht als Kontinuum

Geschlecht wird heute nicht mehr eindeutig biologisch und binär determiniert verstanden. Biologische Merkmale (z. B. Chromosomen, Hormone, äußere und innere Geschlechtsmerkmale) zeigen natürliche Varianz [2], während die Geschlechteridentität, also das persönliche Erleben von Geschlecht, ebenso vielfältig ist. Auch geschlechtsspezifisches Verhalten und Rollenbilder variieren stark über Kulturen und Lebensphasen hinweg. Ein Kontinuumsmodell erkennt an, dass geschlechtliche Realität vielfältig, dynamisch und kontextabhängig ist [19]. Sozial konstruierte Aspekte des Geschlechts – etwa geschlechtsspezifische Sozialisierung oder auch Diskriminierungserfahrungen – können einen maßgeblichen Einfluss auf psychische Gesundheit, Symptompräsentation, Inanspruchnahme von Hilfsangeboten und damit auch den Verlauf von Suchterkrankungen haben.

In diesem Beitrag benutzen wir den Begriff „Geschlecht“ als mehrdimensionale Kategorie, die sowohl auf eine biologische als auch auf eine psychosoziokulturelle Ebene verweist [23]. Sie beinhaltet das biologische Geschlecht („sex“) und das soziale Geschlecht („gender“). Während das biologische Geschlecht körperliche Merkmale wie Anatomie und Hormonhaushalt beschreibt, meint das soziale Geschlecht die gesellschaftlich geprägten Rollen, Normen und Erwartungen, die mit Geschlechtszugehörigkeit verbunden sind [37]. Wir versuchen, mit der integrativen Begrifflichkeit Geschlecht die konstante Wechselwirkung und gegenseitige Bedingung biologischer und sozialer Dimensionen von Geschlecht in den Fokus zu rücken. Eine strikte künstliche Trennung beider Ebenen von Geschlecht ist unzureichend, weil von einer ständigen gegenseitigen Beeinflussung von Biologie und gesellschaftlichen Einflüssen auszugehen ist [23]. Viele der in der Literatur zitierten Studien erfassen jedoch keine spezifischen Merkmale im Hinblick auf das soziale Geschlecht, sondern verwenden „Geschlecht“ meist binär (weiblich/männlich) als biologische Variable. Dabei bleibt oft unklar, inwieweit soziale Rollenbilder, strukturelle Unterschiede oder individuelle Geschlechtsidentitäten tatsächlich berücksichtigt wurden.

Vor diesem Hintergrund verwenden wir im Text überwiegend den Begriff „soziales Geschlecht“ oder „Geschlechterrollen-Selbstkonzepte“, wenn soziale Einflüsse im Mittelpunkt stehen – auch wenn viele Studien formell nur das (biologische) Geschlecht erfassen.

Die Autor*innen weisen darauf hin, dass die Konzentration auf psychosoziale und genderspezifische Aspekte keine Abwertung anderer relevanter Einflussfaktoren darstellt. Faktoren wie genetische Prädispositionen, neurobiologische Grundlagen, familiäre Häufung, Komorbiditäten und soziale Rahmenbedingungen sind ebenso entscheidend für das Verständnis von Alkoholabhängigkeit. Diese Aspekte werden an anderer Stelle ausführlich behandelt.

## Geschlechterrollen als Risikofaktor für Alkoholkonsum

Im Hinblick auf Alkoholkonsum zeigt sich, dass die soziale Konstruktion von Geschlecht und damit verbundene geschlechtsspezifische Erwartungen maßgeblich Einfluss nehmen. Unter Männern gilt Alkoholkonsum nicht nur als gesellschaftlich akzeptiert, sondern wird häufig auch als Ausdruck von Männlichkeit verstanden [18, 38]. Die Orientierung an traditionellen Geschlechternormen kann Männer von der Inanspruchnahme psychischer Hilfe abhalten – was sich unter anderem auch in den höheren Suizidraten widerspiegelt [17]. Rollenerwartungen wie Unabhängigkeit, Stärke und Kontrolle stehen häufig im Widerspruch zu einem offenen Umgang mit emotionalen Belastungen. In diesem Zusammenhang können Suchtmittel als scheinbar selbstbestimmte Form der Bewältigung erscheinen – etwa um Stress, Angst oder Unsicherheit zu regulieren [21, 36].

In männlich geprägten Rollenbildern spielt zudem Leistungsfähigkeit eine zentrale Rolle – beruflich, sexuell, sozial. Wer den Erwartungen nicht entspricht, erlebt häufig Scham und Selbstwertkrisen. In diesen greifen manche Personen, die sich stark mit männlichen Rollenbildern identifizieren, zu Suchtmitteln, insbesondere in leistungsorientierten oder prekären Lebenslagen [1]. Das Konzept der männlichen Depression beschreibt, dass depressive Symptome bei Männern oft anders auftreten als bei Frauen – zum Beispiel eher durch Reizbarkeit, Aggressivität, Rückzug, Suchtverhalten oder gesteigertes Leistungsstreben statt durch Traurigkeit oder Antriebslosigkeit. Diese Ausdrucksformen hängen u. a. mit traditionellen Männlichkeitsnormen zusammen, die emotionale Schwäche tabuisieren und dazu führen können, dass Depressionen bei Männern seltener erkannt und behandelt werden [11]. Eine aktuelle Studie zeigt, dass „maskuline Depression“ mit häufigerem und riskanterem Substanzkonsum, einschließlich Alkohol, einhergeht [38].

Auch Frauen können den Subtyp der maskulinen Depression aufweisen, bei ihnen wurde ebenfalls ein erhöhter und riskanterer Alkoholkonsum festgestellt [38]. Frauen empfinden gleichzeitig häufiger Schuldgefühle nach Alkoholkonsum, was als Folge der stärkeren gesellschaftlichen Stigmatisierung trinkender Frauen gedeutet werden kann [29]. Im Gegensatz zu Normen in der männlichen Sozialisation, sind Konsum und Suchterkrankung schwerer mit den sozialen Erwartungen, die an Frauen gerichtet werden, zu vereinen. Traditionellerweise wird die weibliche Geschlechterrolle eher mit Fürsorge und der Verantwortung für die emotionalen Bedürfnisse anderer assoziiert und kann deshalb auch konfliktvermeidendes Verhalten nahelegen. Dies kann die Nutzung aktiver Bewältigungsstrategien bei Konflikten erschweren und Konsum kann so für Frauen zu einer stillen und ausweichenden Strategie vor negativen Gefühlen und Konflikten genutzt werden [8].

## Geschlechtersensible Versorgung bei Alkoholabhängigkeit: Bedarfe und Entwicklungen im deutschen Suchthilfesystem

Die zunehmende Evidenz für geschlechterspezifische Unterschiede in Ätiologie, Verlauf und Komorbidität alkoholbezogener Störungen hat in den letzten Jahren zur Entwicklung geschlechtersensibler Versorgungsstrukturen in Deutschland beigetragen. Frauen, Männer und LGBTIQ*-Personen unterscheiden sich sowohl hinsichtlich ihrer Konsummuster als auch in der Nutzung, Wahrnehmung und Wirksamkeit von Hilfsangeboten [10, 16]. Das Suchthilfesystem reagiert zunehmend auf diese Heterogenität – jedoch ist die Umsetzung entsprechender Maßnahmen noch uneinheitlich, und insbesondere für LGBTIQ*-Personen bestehen weiterhin deutliche Versorgungslücken. Zur besseren Übersicht listet Tab. 1 im Anhang beispielhafte Angebote geschlechtersensibler Suchtarbeit in Deutschland auf. Die Darstellung umfasst spezifische Einrichtungen für Frauen, Männer sowie trans*, nichtbinäre und queere Personen und zeigt sowohl ambulante als auch stationäre Hilfestrukturen.

### Versorgung von Männern.

Viele Männer nehmen therapeutische Hilfe spät oder gar nicht in Anspruch – nicht zuletzt aufgrund tradierter Männlichkeitsnormen, die Selbstkontrolle, Unabhängigkeit und Emotionsvermeidung betonen [7]. Geschlechtersensible Therapieansätze greifen diese Aspekte gezielt auf, indem sie Raum für die Reflexion von Rollenbildern schaffen und eine Öffnung für emotionale Themen ermöglichen. Die Auseinandersetzung mit Männlichkeitsvorstellungen kann dazu beitragen, die Behandlungsmotivation zu fördern und Rückfällen vorzubeugen. Einzelne Programme adressieren explizit das Spannungsfeld zwischen Selbstbild und Behandlungsmotivation, um die Therapiebereitschaft zu stärken [31]. Die LWL(Landschaftsverband Westfalen-Lippe)-Koordinationsstelle Sucht (LWL-KS) trägt z. B. mit dem Handbuch „Männlichkeiten und Sucht“ [20] sowie den Projektergebnissen „Reine Männersache!? – Suchthilfe in NRW“ zur Sensibilisierung und Qualifizierung von Fachkräften für die Besonderheiten männlicher Suchtbiografien im deutschsprachigen Raum bei (www.lwl-ks.de).

### Versorgung von Frauen.

Frauen mit einer Alkoholabhängigkeit weisen häufiger psychiatrische Komorbiditäten wie Depressionen oder PTBS auf und erleben ihr Suchtverhalten oftmals als besonders schambehaftet [15]. Viele von ihnen bringen Gewalterfahrungen mit, häufig in Form von Partnerschaftsgewalt, was die therapeutische Arbeit zusätzlich erschwert. Gleichzeitig steigt mit einer bestehenden Suchtproblematik auch das Risiko, erneut Opfer von Gewalt zu werden [8]. Diese doppelte Vulnerabilität erfordert spezifische, traumasensible Behandlungsansätze, die Gewalt- und Missbrauchserfahrungen systematisch mitberücksichtigen. Darüber hinaus zeigen sich strukturelle Hürden: Viele Angebote der Suchthilfe sind nicht auf weibliche Lebensrealitäten ausgerichtet. Es fehlt an geschützten Räumen, frauenspezifischen Gruppenangeboten und Einrichtungen mit Betreuungsmöglichkeiten für Kinder.

### Versorgung von LGBTIQ*-Personen.

LGBTIQ*-Personen stehen im deutschen Suchthilfesystem vor besonderen Herausforderungen. Sie sind überdurchschnittlich häufig von Substanzkonsum und -störungen betroffen [27]. Gleichzeitig profitieren sie in geringerem Maße von etablierten psychotherapeutischen Angeboten als cis-heterosexuelle Personen. Hierfür ursächlich sind eine unzureichende Sensibilität gegenüber spezifischen Lebensrealitäten, die fehlende Adressierung gruppenspezifischer Stressoren wie internalisierte Stigmata sowie wiederholte Diskriminierungserfahrungen im Gesundheitswesen [24]. Wie Senreich [32] und weitere Forschende zeigen, berichten LGBTIQ*-Klient*innen in konventionellen Therapiesettings häufig von mangelndem Verständnis und eingeschränktem Sicherheitsgefühl, was die Therapietreue und -wirksamkeit erheblich beeinträchtigen kann. Besonders in der Suchthilfe sowie bei der Behandlung psychischer Komorbiditäten werden LGBTIQ*-spezifische Stressoren in klassischen Therapieansätzen oft nur unzureichend berücksichtigt [32].

Angesichts dieser komplexen Belastungsfaktoren ist die Entwicklung identitätsaffirmativer und strukturell inklusiver Behandlungsansätze dringend erforderlich. Dazu gehört u. a. der bewusste und respektvolle Umgang mit Namen, Pronomen und Identitäten sowie die Auseinandersetzung mit cis-normativen Rollenvorstellungen. Insgesamt zeigt sich ein positiver Entwicklungstrend hin zu geschlechtsspezifisch differenzierten und inklusiven Therapieangeboten in Deutschland (siehe entsprechende Fördermaßnahmen des Bundesministeriums und der Bundestherapeutenkammer [4, 5]).

Für die Praxis ergeben sich daraus konkrete Implikationen: Die Einbindung gendersensibler Anamneseinstrumente [34], die Kooperation mit spezialisierten Einrichtungen sowie interdisziplinäre Netzwerke zur Begleitung vulnerabler Gruppen sind zentrale Elemente einer zeitgemäßen suchtmedizinischen Versorgung. Die in Tab. 1 und 2 im Anhang dargestellten Übersichten verstehen sich dabei als ergänzende Orientierungshilfen, um Fachkräften den Zugang zu konkreten Angeboten und praxisrelevanten Ressourcen zu erleichtern.

## Komplexe Interaktionen: Geschlechtsidentität, Alkoholabhängigkeit und Partnerschaftsgewalt

Alkoholabhängigkeit und Partnerschaftsgewalt treten häufig gemeinsam auf – Alkohol wirkt dabei nicht nur als Risikofaktor für gewalttätiges Verhalten, sondern wird von Betroffenen teils auch als Mittel zur Bewältigung eigener Gewalt- oder Ohnmachtserfahrungen eingesetzt. In bestimmten Männlichkeitsbildern, die Kontrolle, Dominanz und emotionale Unverletzbarkeit betonen, kann Alkohol eine funktionale Rolle einnehmen – etwa zur Kompensation von Unsicherheit, Versagensängsten oder dem Verlust traditioneller Statussymbole wie Erwerbstätigkeit [26]. Zudem kann Alkohol im Sinne von „liquid courage“ gewaltbereites Verhalten begünstigen, indem er Hemmschwellen senkt und das Gefühl von Risikobereitschaft oder Unverwundbarkeit verstärkt [26]. Partnerschaftsgewalt tritt besonders häufig im Kontext von Intoxikation oder Craving auf, wird jedoch auch beeinflusst durch emotionale Faktoren wie sexuelle Eifersucht oder den subjektiv wahrgenommenen Widerstand gegen männliche Autorität [13].

Laut polizeilicher Kriminalstatistik von 2023 waren 80 % der Betroffenen von Partnerschaftsgewalt weiblich, 78 % der Tatverdächtigen männlich – und in 23 % der Fälle spielte Alkohol eine Rolle [3]. In suchttherapeutischen Settings berichtet rund die Hälfte der alkoholabhängigen Männer, im vergangenen Jahr Gewalt gegenüber ihrer Partnerin ausgeübt zu haben [12]. Auch trans*-Personen sind überdurchschnittlich häufig betroffen – mit einem direkten Zusammenhang zwischen Gewalterfahrungen und Substanzkonsum [25]. Eine schwedische Längsschnittstudie zeigt zudem, dass Männer mit Substanzkonsumstörungen – als Haupt- oder Nebendiagnose – im Vergleich zu ihren nichtsubstanzabhängigen Geschwistern, ein signifikant erhöhtes Risiko für die Ausübung von Partnerschaftsgewalt aufweisen [39]. Darüber hinaus gelten Gewalterfahrungen in der Kindheit als gut belegter Risikofaktor für spätere Suchtentwicklungen [9]. Gleichzeitig ist wichtig zu betonen, dass auch Männer Opfer von Partnerschaftsgewalt sein können – unabhängig vom Geschlecht der gewaltausübenden Person. Eine umfassende und sensible Erfassung solcher Erfahrungen muss daher geschlechterübergreifend und vorurteilsfrei erfolgen.

Diese Befunde unterstreichen die Notwendigkeit, Partnerschaftsgewalt systematisch in der Suchthilfe zu adressieren (weiterführende Ressourcen zum Umgang mit Partnerschaftsgewalt finden sich im Anhang, Tab. 2). Therapeutische Fachkräfte sollten gezielt darin geschult werden, Gewaltbelastungen sensibel zu erkennen, professionell anzusprechen und adäquat darauf zu reagieren.

## Limitationen

Die Auswahl der in diesem Beitrag dargestellten Studien erfolgte exemplarisch und basierend auf der Expertise des Autor*innenteams. Grundlage hierfür war die konzeptionelle Relevanz der Arbeiten im Hinblick auf psychosoziale und geschlechtsspezifische Fragestellungen, nicht jedoch ein strukturierter Such- oder Auswahlprozess im Sinne systematischer Übersichtsarbeiten. Eine Bewertung der methodischen Qualität der einzelnen Studien wurde nicht vorgenommen.

Ein inhaltlicher Fokus lag unter anderem auf der Darstellung partnerschaftlicher Gewalt, wobei aufgrund ihrer Häufigkeit insbesondere Gewalt gegenüber Frauen thematisiert wurde. Potenzielle Gewaltbetroffene anderer Geschlechtsidentitäten – etwa männliche oder nichtbinäre Opfer – wurden nicht systematisch berücksichtigt.

Internationale Forschungsarbeiten zur Rolle von Geschlechterrollen und gesellschaftlichen Kontexten im Zusammenhang mit Alkoholabhängigkeit wurden nur exemplarisch einbezogen. Ein umfassender kulturvergleichender Zugang lag außerhalb des Rahmens dieses Beitrags, stellt jedoch ein relevantes Feld für zukünftige Untersuchungen dar.

## Schlussfolgerung und Ausblick

Geschlechterrollen stellen einen zentralen Einflussfaktor für das Suchtverhalten dar. Ihre Erfassung in der Forschung ist essenziell, da sie sowohl den Konsum selbst als auch die Inanspruchnahme und Wirksamkeit therapeutischer Angebote maßgeblich mitbestimmen (für Empfehlungen zu Erfassung des sozialen und biologischen Geschlechts siehe Empfehlungen der Arbeitsgruppe Geschlecht und Psychische Gesundheit des Deutschen Zentrums für Psychische Gesundheit in dieser Ausgabe von *Der Nervenarzt*).

Um wirksam intervenieren zu können, brauchen Betroffene individuelle, geschlechtssensible Behandlungsangebote – häufig mit Fokus auf Traumaverarbeitung, psychosoziale Belastungen sowie niedrigschwellige, stigmafreie Zugänge. Insbesondere rigide traditionelle Männlichkeitsnormen wirken in diesem Kontext doppelt problematisch: Sie fördern riskanten Alkoholkonsum, erschweren den emotionalen Ausdruck und verzögern oder verhindern die frühzeitige Suche nach Hilfe. Damit stellen sie einen relevanten Risikofaktor sowohl für die Entwicklung als auch für die Aufrechterhaltung von Suchtverhalten dar.

Geschlechtersensible Suchthilfe muss diese Zusammenhänge offen thematisieren, um passgenaue und wirksame Angebote zu schaffen – insbesondere für Männer, deren Problemlagen im bestehenden System häufig nicht adäquat abgebildet werden.

Auch Behandler*innen sind gefordert, ihre eigenen geschlechtsspezifischen Haltungen zu reflektieren und das Thema Männlichkeit und die Implikationen rigider Geschlechterrollen-Selbstkonzepte im therapeutischen Kontext aktiv und beziehungsorientiert anzusprechen. Ein flexibler, nichtnormativer Umgang mit Geschlechterrollen kann wesentlich zur Entstigmatisierung und Heilung beitragen.

Letztlich ist auch eine gesamtgesellschaftliche Auseinandersetzung mit den gesundheitlichen Folgen rigider Geschlechterbilder notwendig – nicht zuletzt angesichts der deutlich höheren Prävalenz alkoholbezogener Störungen und Suizide bei Männern.

## Fazit für die Praxis

- Epidemiologische Daten zeigen signifikante Unterschiede zwischen den Geschlechtern hinsichtlich Prävalenz, Konsummuster und Komorbiditäten bei Alkoholabhängigkeit. Diese Unterschiede sind nicht nur biologisch bedingt, sondern spiegeln u. a. auch soziale Konstruktionen von Geschlecht und geschlechtsspezifische Rollenerwartungen wider.
- Geschlechtersensible Suchthilfe muss diese Zusammenhänge offen thematisieren, um passgenaue und wirksame Angebote zu schaffen – insbesondere für Männer, deren Problemlagen im bestehenden System häufig nicht adäquat abgebildet werden.

## Supplementary Information

Tabellen online

## Footnotes

1 Der Begriff LGBTIQ* steht für die englischen Begriffe lesbisch, schwul, bisexuell, trans*, inter* und queer. Er umfasst eine Vielzahl sexueller und geschlechtlicher Identitäten, die jenseits hetero- und cis-normativer Vorstellungen liegen. Das Sternchen (*) signalisiert, dass viele weitere Identitäten und Erfahrungen eingeschlossen sind.

2 Cis-Geschlechtlichkeit bedeutet, dass eine Person sich mit dem bei der Geburt zugewiesenen Geschlecht identifiziert.
