# Supplementary material for: Impact of gender roles on alcohol dependence
Source: Nervenarzt. 2025 Sep 29;97(1):27–33. [Article in German] doi: 10.1007/s00115-025-01904-9 (PMC12808160; doi:10.1007/s00115-025-01904-9)
Supplement: Supplementary file 1 — Tabellen online [file 115_2025_1904_MOESM1_ESM.pdf]

Anhang

Tabelle e1: Übersicht gendersensibler Angebote bei Alkoholabhängigkeit in Deutschland

Hinweise:

- Regionale Unterschiede: Die Verfügbarkeit kann stark variieren, besonders im ländlichen Raum.
- Fehlende strukturelle Verankerung: Insbesondere trans* und nicht-binäre Personen sind auf Einzelinitiativen und Modellprojekte angewiesen.
- Bedarf an Fortbildung: Zahlreiche Fachstellen (Bundesinstitut für öffentliche Gesundheit, BIÖG) bieten Schulungsmaterialien für gendersensible Suchtarbeit an.

Übersicht gendersensibler Angebote bei Alkoholabhängigkeit in Deutschland

**Zielgruppe**	**Einrichtung/Projekt**	**Angebotsform**	**Ort/Träger**	**Besonderheiten/Fokus**
Frauen	BELLA DONNA	Beratung, Therapie, Krisenintervention	Essen / BELLA DONNA e.V.	Spezialisierung auf frauenspezifische Suchtarbeit, inklusive Angebote in Justizvollzugsanstalten für Frauen
Frauen	FrauSuchtZukunft	Beratung, ambulante Therapie, Krisenintervention	Berlin / FrauSuchtZukunft e.V.	Psychosoziale Betreuung, Beratung und Clearing, Kriseninterventionen, ambulante Suchttherapie, Besuche und Beratungen in der JVA für Frauen
Frauen	LAGAYA	Beratung, ambulante Therapie, Betreutes Wohnen	Stuttgart / LAGAYA e.V.	Frauenspezifische Ansätze in der Suchtarbeit, Angebote für Betreutes Wohnen, ambulante Beratung von Mädchen
Frauen	Beratungsstelle Frauenperspektiven	Ambulante Suchttherapie, Nachsorge, Beratung	Hamburg / Frauenperspektiven e.V.	Entwicklung von Perspektiven für suchtmittelabhängige Frauen, ambulante Nachsorge, Beratung und Prävention für Mädchen zu Sucht und Drogen
Frauen	FrauSuchtHilfe	Aufklärung, Selbsthilfe, Öffentlichkeitsarbeit	Deutschlandweit / Deutscher Frauenbund	Fokus auf Entstigmatisierung, Aufklärung über frauenspezifische Suchtformen, Unterstützung beim Wiederfinden von Selbstbewusstsein.
Frauen	Therapieverbund Ludwigsmühle	Stationäre & ambulante Behandlung	Rheinland-Pfalz	Traumaorientierte Suchttherapie für Frauen
Frauen	FrauenSuchtGesundheit e.V.	Beratung & Vernetzung	Hamburg	Multiprofessionelle Beratung, auch zu Gewalt und Trauma
Frauen	Fachklinik Münzesheim	Stationär	Baden-Württemberg	Suchtbehandlung mit frauenspezifischem Schwerpunkt

Frauen	Frauenberatungsstellen (div.)	Niedrigschwellig / psychosozial	Regional	Integrieren Suchtprävention und Krisenintervention
Frauen	Pro familia, Caritas, Diakonie	Amb. Suchthilfe mit Frauensprechstunden	Bundesweit	Gendersensibler Zugang über multiprofessionelle Kooperation
Männer	AWO Suchthilfe Thüringen (z. B. Suchtberatungsstelle Erfurt)	Ambulant, männerspezifische Gruppen	Thüringen	Männlichkeitsnormen, emotionaler Zugang
Männer	Fachklinik Vielbach	Stationär	Rheinland-Pfalz	Therapieangebote für Männer mit Fokus auf Rollenerwartungen
Männer	Männerbüros (z. B. Nürnberg, Wien – übertragbar)	Beratung, Gruppen	Lokal (Modellprojekte)	Bearbeitung von Sucht als Coping im Männlichkeitskontext
Männer	LWL-Koordinationsstelle Sucht	Forschung, Beratungskonzepte	Münster / LWL	Entwicklung von Konzepten zur gendersensiblen Suchtarbeit für Männer
Männer	Fachklinik Möhringsburg	Stationäre Entzugsbehandlung mit genderspezifischer Therapie	Klinikum Osnabrück	Geschlechtsspezifische Gruppen, Thematisierung männlicher Rollenbilder und deren Einfluss auf Suchtverhalten
Homo- und bisexuelle Menschen	SHALK NRW	Selbsthilfegruppen	NRW / SHALK NRW	Netzwerk für homo- und bisexuelle Menschen mit Suchterkrankung, aktiv in zehn Städten
Trans*, nicht-binäre Personen	4be TransSuchthilfe	Beratung, Gruppenangebote, Fortbildungen	Hamburg / 4be TransSuchthilfe	Spezialisierte Angebote für trans*, nicht-binäre und genderdiverse Menschen, Peer-Beratung
Trans*, nicht-binäre Personen	Schwulenberatung Berlin – Transvisible	Ambulante Gruppen & Beratung	Berlin	Transaffirmative Gruppenarbeit, Peer-Support, Sucht & Identität
Trans*, nicht-binäre Personen	Berliner Zentrum für trans* und inter* Gesundheit	Beratung, medizinisch-sozial	Charité Berlin (Kooperationen)	Inklusion von Suchtfragestellungen bei trans* Gesundheitsfragen
Trans*, nicht-binäre Personen	aidshilfe NRW / Deutsche Aidshilfe	Schulungen, Beratungsnetzwerke	Bundesweit	LGBTQ*-inklusive Suchtprävention, Fortbildung für Fachkräfte
Trans*, nicht-binäre Personen	Wildwasser Berlin / Köln	Schwerpunkt sexualisierte Gewalt	Berlin / Köln	Für trans* FLINTA mit Sucht- und Traumaerfahrungen

Tabelle e2: Ressourcen zur Weiterbildung und praktischen Anwendung bei Partnerschaftsgewalt (Auswahl)

BIG Hotline, telefonische Beratung bei Häuslicher Gewalt – rund um die Uhr erreichbar, bei Bedarf kann in andere Sprachen übersetzt werden	030 6110300<br>https://www.hilfetelefon.de
Regelmäßige Fortbildungen zur Versorgung gewaltbetroffener Patient*innen	https://www.signal-intervention.de/fortbildungen-und-qualifizierung
klinisches Handbuch zur Versorgung gewaltbetroffener Frauen der WHO, in mehreren Sprachen (auch deutsch) verfügbar	https://www.who.int/publications/i/item/WHO-RHR-14.26
Beratungsstellen für Täter*innen	https://www.bag-taeterarbeit.de/beratungsstellen/
Onlinetool zur Risikoeinschätzung (deutsch) - Ontario Domestic Assault Risk Assessment (ODARA)	https://www.knfp.ch/de/prognosetools/odara
Handbuch zur sicheren Arbeit mit männlichen Tätern in Suchtbehandlungssettings (englisch)	https://coercivecontrol.ripfa.org.uk/wp-content/uploads/A_framework_for_working_safely_and_effectively_with_perpetrators.pdf
